# Recognizing the Benign Behind Worrisome Histology: A Case Report of Proliferative Fasciitis

**DOI:** 10.3390/reports9030271

**Published:** 2026-08-14

**Authors:** Catalin-Bogdan Satala, Valerica Valentin Zaharia, Alina-Mihaela Gurau, Cristina-Mihaela Popescu, Robert Daniel Ciortan, Daniela Mihalache

**Affiliations:** 1 Faculty of Medicine and Pharmacy, Medical and Pharmaceutical Research Center, “Dunărea de Jos” University of Galati, 800008 Galati, Romania; catalin.satala@ugal.ro (C.-B.S.); cristina.popescu@ugal.ro (C.-M.P.); robert.ciortan@ugal.ro (R.D.C.); daniela.mihalache@ugal.ro (D.M.); 2 Department of Pathology, Clinical County Emergency Hospital Braila, 810325 Braila, Romania; 3 The School for Doctoral Studies in Biomedical Sciences, “Dunărea de Jos” University of Galati, 800008 Galati, Romania

**Keywords:** case report, proliferative fasciitis, spindle cell lesion, *FOS* rearrangement, fibroblastic/myofibroblastic proliferation, soft tissue pathology, immunohistochemistry

## Abstract

**Background and Clinical Significance**: Proliferative fasciitis (PF) is an infrequent benign fibroblastic/myofibroblastic proliferation that may closely resemble a soft tissue sarcoma, creating a diagnostic dilemma out of proportion to its biological behaviour. Because no single clinical, histological or immunohistochemical feature is diagnostic, accurate classification depends on the integration of complementary findings. We describe a challenging case of PF involving the lower leg and present a practical clinicopathological approach to its evaluation. **Case Presentation**: A 34-year-old man presented with a painless subcutaneous nodule on the lateral aspect of the left lower leg, discovered incidentally. Clinical examination suggested a benign superficial soft-tissue lesion, and because no features raised suspicion for malignancy, complete excision was performed without preoperative imaging. Gross examination revealed a 1.9 × 1.6 × 0.7 cm fascial-based lesion composed of spindle cells and scattered ganglion-like cells within a variably myxoid stroma. Focal nuclear pleomorphism, typical mitotic activity (2 mitoses/10 high-power fields), and limited extension into adjacent adipose tissue broadened the differential diagnosis. Immunohistochemistry demonstrated focal SMA positivity, weak focal desmin and S100 expression, absence of CD31 and CD34 staining, and a low Ki-67 proliferative index (approximately 2–3%). Negative surgical margins, together with integration of the clinical presentation, gross findings, histomorphology, and immunophenotype, supported the diagnosis of proliferative fasciitis. The patient remains free of local recurrence four months after surgery. **Conclusions**: PF should be considered in the differential diagnosis of superficial spindle-cell proliferations showing deceptively aggressive histological features. Careful clinicopathological correlation remains the cornerstone of diagnosis and helps distinguish this benign entity from its malignant mimics. The clinicopathological framework proposed in this report may assist pathologists in the systematic evaluation of similar diagnostically challenging lesions.

## 1. Introduction and Clinical Significance

Soft tissue pathology remains one of the few areas of surgical pathology in which morphology can still challenge biology. Despite remarkable advances in immunohistochemistry and molecular diagnostics, the distinction between reactive fibroblastic proliferations and true mesenchymal neoplasms continues to rely, in many cases, on careful interpretation of architectural and cytological patterns rather than on a single defining ancillary test [1,2]. This challenge is particularly evident in the group of benign pseudosarcomatous proliferations, where lesions with entirely indolent biological behavior may display an alarming combination of rapid clinical growth, marked cellularity, mitotic activity, cytological atypia, and infiltrative-appearing borders, features traditionally regarded as hallmarks of malignancy. Consequently, these lesions remain a well-recognized source of diagnostic error and may lead to unnecessary aggressive treatment when interpreted outside their appropriate clinicopathological context [1,3].

Proliferative fasciitis (PF), first described by Chung and Enzinger in 1975, represents a distinctive benign pseudosarcomatous fibroblastic-myofibroblastic proliferation that typically arises within the superficial fascia and subcutaneous tissues of adults [4]. Although uncommon, PF occupies a unique position among soft tissue lesions because its microscopic appearance frequently appears disproportionate to its biological behavior. Clinically, it often presents as a rapidly enlarging solitary mass, raising immediate concern for a sarcoma [5,6]. Histologically, the lesion is classically composed of spindle-shaped myofibroblasts admixed with characteristic ganglion-like cells within a variably collagenous and focally myxoid stroma. However, the morphological spectrum of PF extends beyond this conventional description, and diagnostically challenging cases may exhibit pronounced hypercellularity, conspicuous nucleoli, readily identifiable mitotic figures, focal pleomorphism, stromal myxoid change, hemorrhagic foci, and infiltrative-appearing extension into adjacent adipose tissue, substantially expanding the differential diagnosis [6].

Perhaps the most important diagnostic characteristic of PF is that no individual microscopic feature is pathognomonic. The diagnosis is established neither by the presence of ganglion-like cells alone, nor by a characteristic immunophenotype, but by recognizing the overall constellation of histological findings within the appropriate clinical setting. Features that, when viewed in isolation, would reasonably raise suspicion for malignancy may collectively define a benign reactive process. Accordingly, accurate diagnosis requires a pattern-based approach that integrates tissue architecture, stromal composition, cytological characteristics, and clinical presentation while systematically excluding malignant spindle cell neoplasms with overlapping morphology [5,6].

The practical implications of this distinction are considerable. Overdiagnosis may result in unnecessarily aggressive surgical management, additional staging procedures, prolonged oncological surveillance, and significant psychological burden for the patient. Conversely, excessive diagnostic conservatism may delay the recognition of genuine sarcomas. For this reason, awareness of the full histopathological spectrum of PF remains essential for surgical pathologists, particularly when evaluating hypercellular spindle cell proliferations with pseudosarcomatous features [5,6].

In the present report, we describe a case of PF arising in the lower extremity of a young adult male that posed a significant diagnostic challenge because of its unusual combination of hypercellularity, cytological atypia, and infiltrative-appearing growth pattern, closely simulating a malignant spindle cell neoplasm. Using this case as a diagnostic framework, we review the morphological spectrum of PF, discuss its principal histopathological mimics, and emphasize the morphological clues that remain critical for distinguishing this benign pseudosarcomatous proliferation from its malignant counterparts.

## 2. Case Presentation

A 34-year-old man presented with a painless subcutaneous nodule on the lateral aspect of his left lower leg, which he had noticed incidentally while showering approximately two weeks before presentation. The lesion had remained stable in size and was not associated with overlying skin changes, tenderness, or functional impairment. The patient recalled a possible minor blunt trauma to the same region sustained during a recreational tennis match approximately two months earlier; however, the temporal relationship between this event and the subsequent development of the lesion remained uncertain.

His medical history was significant only for well-controlled arterial hypertension. He had no previous history of neoplastic disease, connective tissue disorders or autoimmune conditions. Family history was negative for malignancy, and he was a lifelong non-smoker.

Physical examination demonstrated a solitary, elastic, non-tender subcutaneous nodule measuring 1.9 cm in greatest dimension. The lesion was relatively mobile but showed slight adherence to the underlying fascial plane. No abnormalities of the overlying skin were identified, and regional lymph nodes were not enlarged.

Routine laboratory investigations, including complete blood count, C-reactive protein, erythrocyte sedimentation rate, renal and liver function tests, serum electrolytes, coagulation profile and fasting blood glucose, were all within normal reference ranges.

Based on its accessible location, small size, absence of pain, and lack of clinically aggressive features, the lesion was considered most consistent with a benign superficial soft tissue process. The preoperative clinical differential diagnosis included a benign fibrous or fascial lesion, epidermal inclusion cyst, lipoma, and organized post-traumatic soft tissue change. Because malignancy was considered unlikely, complete excision was selected as both the diagnostic and therapeutic procedure.

### 2.1. Gross Pathological Findings

**Figure 1:**
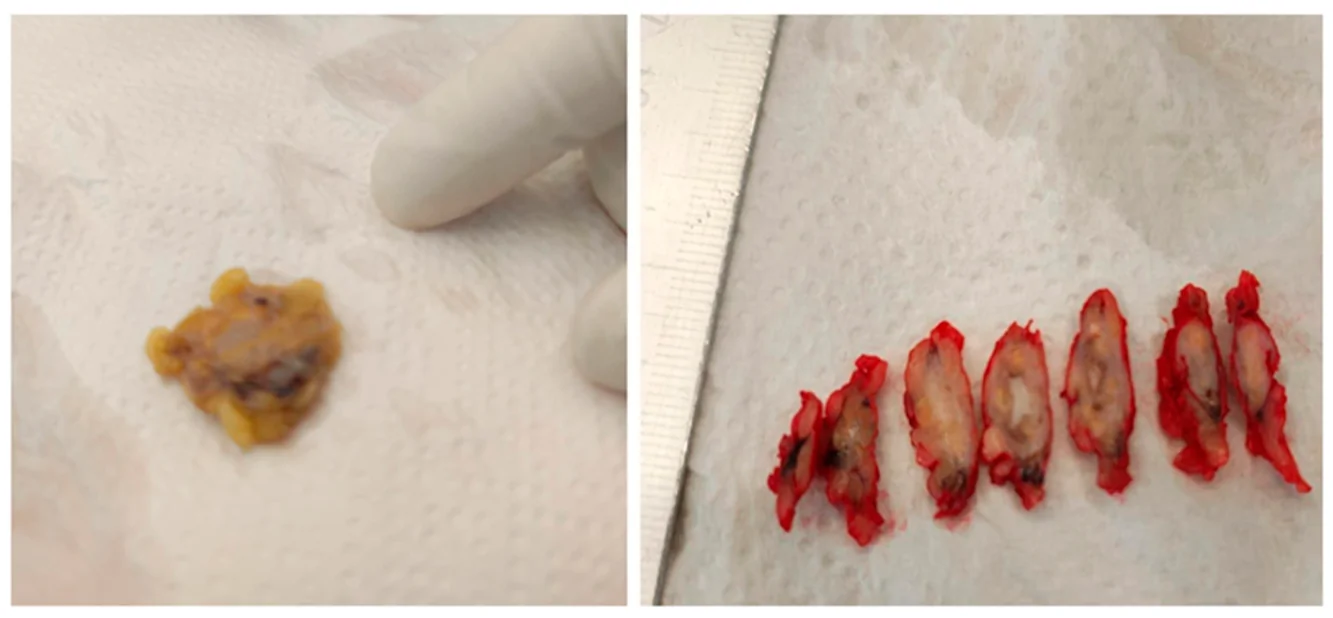
Gross pathological appearance. Relatively well-demarcated, non-encapsulated subcutaneous lesion with a predominantly solid grey-white cut surface and peripheral adipose tissue showing fat necrosis. No gross haemorrhage or tumor necrosis is identified.

The specimen was received unoriented and consisted of a nodular soft tissue lesion measuring 1.9 × 1.6 × 0.7 cm, located immediately above the superficial fascia, to which it was focally adherent. The external surface was smooth, glistening and grey-white, without evidence of encapsulation. Serial sectioning demonstrated a predominantly solid, grey-white and yellowish cut surface with a centrally located translucent area. Several minute violaceous foci were present within the lesion. Small fragments of surrounding adipose tissue were attached at the periphery and showed focal yellow discoloration corresponding to fat necrosis. No gross haemorrhage, cystic degeneration or coagulative necrosis was identified (Figure 1).

### 2.2. Histopathological Findings

Microscopic examination demonstrated a highly cellular fibroblastic and myofibroblastic proliferation centered within the subcutaneous tissue in close relationship to the superficial fascia, which was focally incorporated at the lesion periphery. At scanning magnification, the lesion appeared relatively well delineated but lacked a true capsule. Focal irregular extension into the surrounding adipose tissue imparted a pseudoinfiltrative appearance without evidence of destructive growth. At the periphery, focal adipose tissue injury was associated with a mild lipogranulomatous reaction composed of lipid-laden histiocytes and occasional multinucleated giant cells. These findings were interpreted as secondary reactive changes rather than evidence of infiltrative growth. The spindle-cell proliferation displayed subtle architectural heterogeneity, alternating between diffuse hypercellular areas, short ill-defined fascicles and focal tissue culture-like regions. Towards the center of the lesion, these areas gradually transitioned into a looser myxoid stroma containing numerous extravasated erythrocytes.

**Figure 2:**
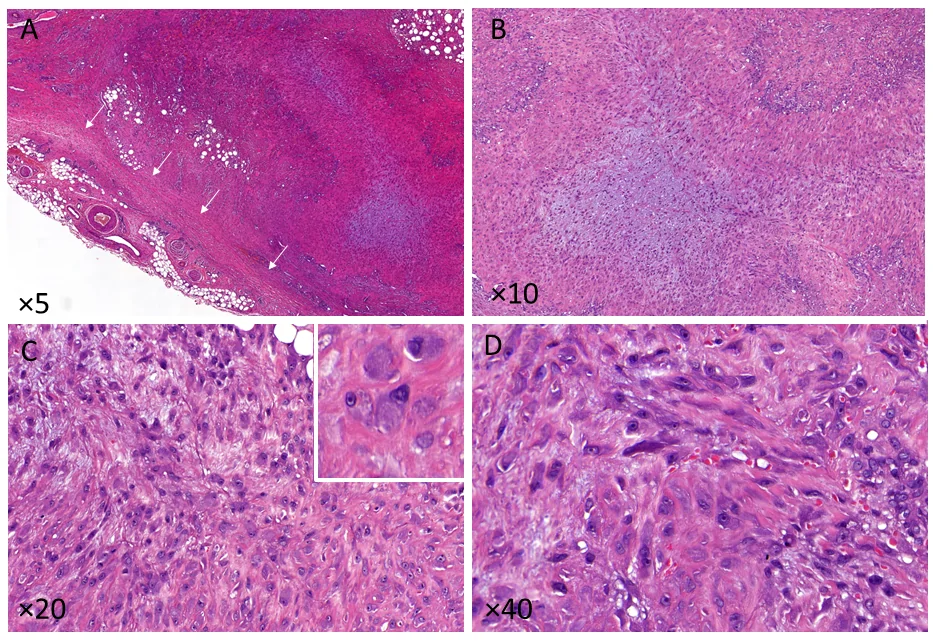
Histopathological features of proliferative fasciitis. (**A**) Low-power view showing a subcutaneous fibroblastic-myofibroblastic proliferation with focal extension into adjacent adipose tissue, adjacent to fascia (arrows). (**B**) Alternating hypercellular and myxoid areas. (**C**) Characteristic ganglion-like cells within the spindle-cell proliferation (inset). (**D**) Mild cytological atypia without atypical mitoses or tumour necrosis.

The lesional cells consisted predominantly of plump spindle cells with eosinophilic cytoplasm and nuclei showing evident nucleoli. Interspersed throughout the proliferation were scattered large polygonal cells with abundant amphophilic to eosinophilic cytoplasm, vesicular nuclei and prominent eosinophilic nucleoli, imparting the characteristic ganglion-like appearance. Mild nuclear pleomorphism was present but remained focal. Occasional osteoclast-like multinucleated giant cells were identified throughout the lesion. Mitotic activity averaged 2 mitoses per 10 high-power fields, and all mitotic figures were morphologically typical. Neither atypical mitotic figures nor coagulative tumour necrosis was identified (Figure 2).

### 2.3. Immunohistochemical Profile

**Figure 3:**
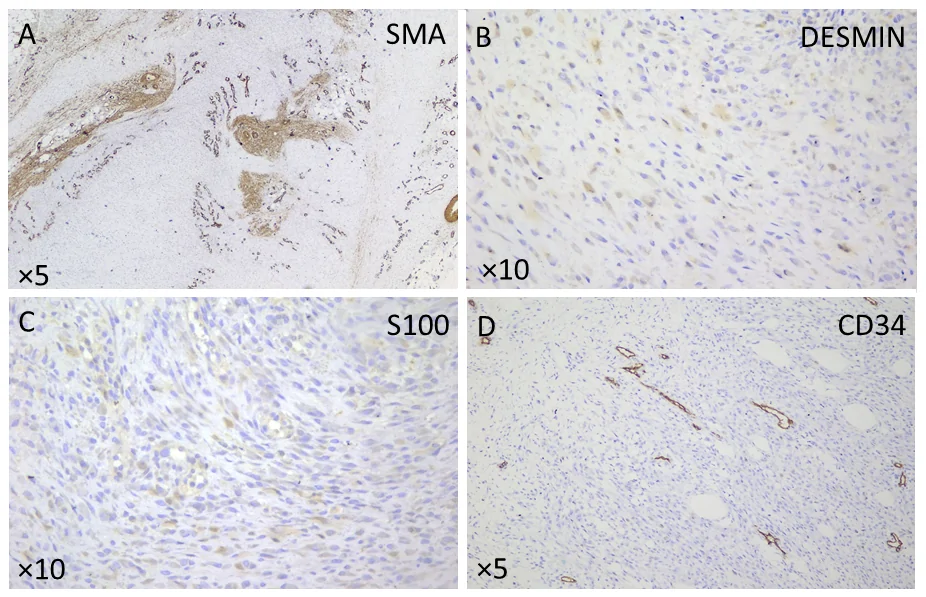
Immunohistochemical findings. (**A**) Focal smooth muscle actin (SMA) expression showing a tram-track pattern. (**B**) Weak focal desmin positivity. (**C**) Weak focal S100 expression, most evident in scattered ganglion-like cells. (**D**) Absence of CD34 expression in lesional cells with preserved staining of vascular endothelial cells as internal positive controls. Immunostaining was assessed qualitatively during routine diagnostic evaluation; no semiquantitative scoring system was applied.

Immunohistochemical analysis demonstrated focal smooth muscle actin (SMA) expression, predominantly involving the peripheral spindle-cell component in a characteristic tram-track distribution. Desmin showed weak focal cytoplasmic staining, while S100 demonstrated weak focal positivity, predominantly within scattered ganglion-like cells and occasional spindle cells. The Ki-67 proliferative index was approximately 2–3% of lesional cells. CD34 and CD31 were negative in both the spindle-cell and polygonal cell populations. The immunohistochemical panel was selected to support myofibroblastic differentiation and to assist in excluding the principal histological mimics. Nevertheless, the final diagnosis relied on integration of the immunophenotypic findings with the characteristic morphology and clinicopathological context (Figure 3).

Based on the histomorphological appearance, the principal pathological differential diagnoses included nodular fasciitis, proliferative myositis, low-grade myofibroblastic sarcoma and undifferentiated pleomorphic sarcoma. Correlation of the microscopic findings with the immunophenotype, anatomical location and clinical presentation favoured a reactive myofibroblastic proliferation, and a final diagnosis of proliferative fasciitis was established.

Histological examination confirmed complete excision with negative surgical margins. No additional surgical treatment was considered necessary following histopathological diagnosis, and the patient was managed by clinical surveillance. The postoperative course was uneventful, with no procedure-related adverse events or complications. At four months of follow-up, he remained asymptomatic, with no clinical evidence of local recurrence. However, the duration of follow-up is relatively short and does not allow definitive assessment of long-term outcome.

### 2.4. Patient Perspective

**Figure 4:**
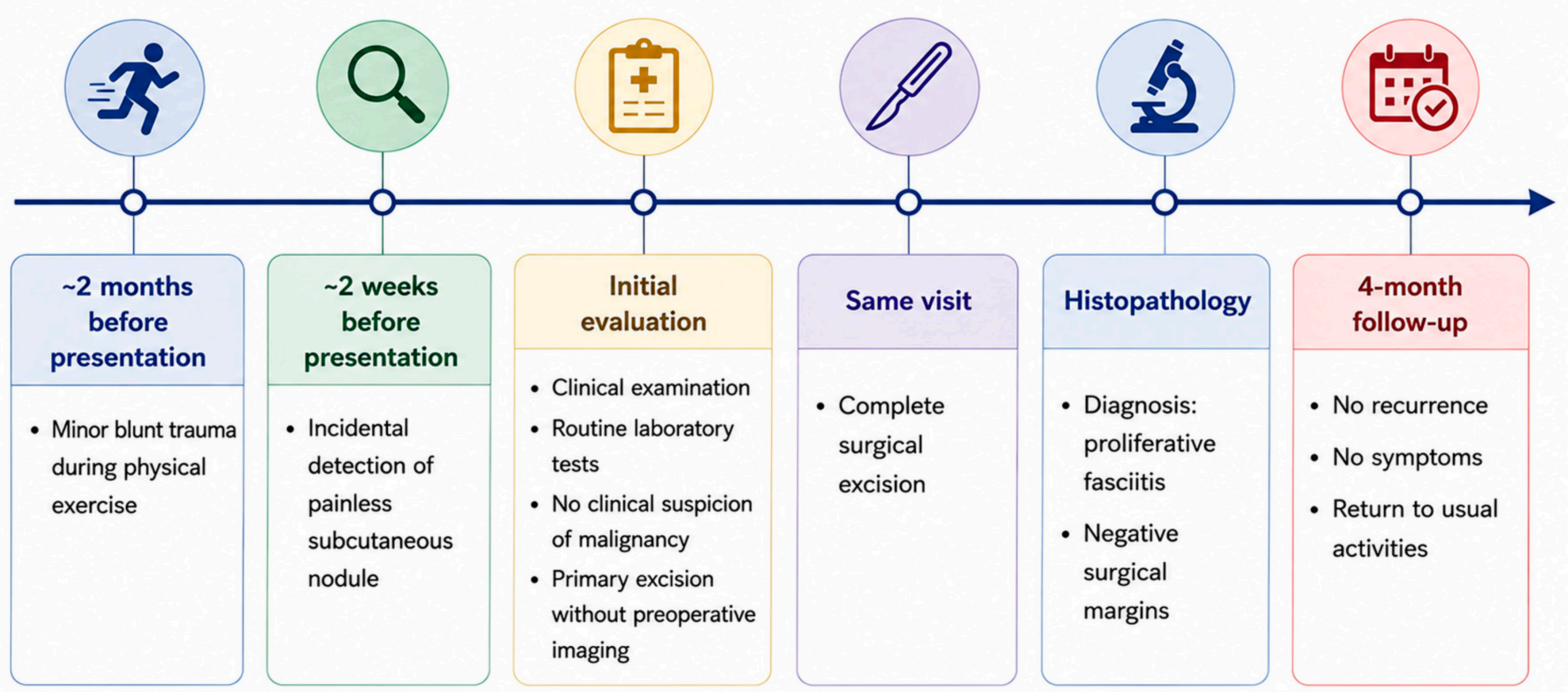
Clinical timeline summarizing the patient’s presentation, diagnostic evaluation, surgical treatment, histopathological diagnosis, and postoperative follow-up.

The patient reported complete resolution of symptoms following surgical excision. He returned to his usual daily and recreational activities without functional limitations. The patient’s clinical course, from initial presentation to postoperative follow-up, is summarized in Figure 4.

## 3. Discussion

Few benign soft tissue lesions create as much diagnostic hesitation as proliferative fasciitis when examined for the first time under the microscope. The difficulty does not arise because the lesion lacks defining characteristics, but because several of its expected histological features closely resemble changes that are traditionally associated with malignancy. Nearly five decades after Chung and Enzinger first characterized proliferative fasciitis as a distinctive pseudosarcomatous fibroblastic proliferation, this paradox continues to shape everyday diagnostic practice. Although subsequent clinicopathological studies have broadened the recognized histological spectrum, they have also confirmed that no two cases appear entirely alike, particularly when the relative prominence of individual microscopic findings varies across the lesion [4,5].

The current case falls within this diagnostically demanding end of the spectrum. None of its individual features would be considered exceptional in isolation. Increased cellularity, focal nuclear pleomorphism, readily identifiable mitotic figures, myxoid stromal change and irregular extension into adipose tissue have all been documented in previous series. What complicated the diagnosis was their coexistence within a relatively small lesion, creating an appearance that, at least initially, seemed disproportionate to its benign clinical presentation. This point is clinically relevant because it reflects a situation encountered far more frequently in routine reporting than in textbook illustrations, where classic examples inevitably dominate [5,7,8].

Although proliferative fasciitis is classically described as a rapidly enlarging lesion, our patient reported no appreciable increase in lesion size during the two weeks between its incidental discovery and surgical excision. This observation highlights the clinical heterogeneity of proliferative fasciitis and suggests that rapid enlargement, although common, should not be regarded as an indispensable diagnostic feature. Lesions detected incidentally at an early stage may remain clinically stable over a short observation period while still displaying the characteristic histopathological features of proliferative fasciitis.

Published descriptions often emphasize the characteristic ganglion-like cells and tissue culture-like appearance as the defining hallmarks of proliferative fasciitis. These features are undoubtedly important, yet they represent only part of a broader histological landscape. In routine specimens, the distribution of characteristic findings is rarely uniform. Some areas may display the expected reactive appearance almost immediately, whereas others are dominated by hypercellularity or stromal alterations that broaden the differential diagnosis considerably. The diagnosis usually becomes clear only after the lesion has been examined at different magnifications and across multiple sections, rather than from a single microscopic finding [6,8].

Recent molecular findings provide additional insight into this process. The demonstration of recurrent *FOS* rearrangements in proliferative fasciitis and proliferative myositis has challenged the traditional view of these lesions as purely reactive proliferations, suggesting a transient clonal process with a distinctive molecular background. At the same time, these discoveries have had remarkably little impact on routine diagnostic practice. In most pathology laboratories, the diagnosis is still established without molecular confirmation and continues to rely on the correlation of clinical information with conventional histopathological assessment. Molecular findings have therefore refined our understanding of the biology of proliferative fasciitis far more than they have altered its day-to-day diagnosis [5,9].

Microscopic interpretation is influenced not only by the presence of individual histological findings, but also by the order in which they are recognized. Some microscopic findings naturally attract attention more than others. In spindle-cell proliferations, increased cellularity, conspicuous nucleoli or mitotic activity may initially shift the differential diagnosis toward sarcoma before the overall architecture has been fully assessed. Large nucleoli, increased cellularity or mitotic activity are visually striking and naturally prompt closer scrutiny, whereas more reassuring findings tend to emerge only after the lesion has been examined more broadly. This sequence is rarely discussed explicitly, yet it probably explains why proliferative fasciitis continues to be overcalled despite its well-established clinicopathological profile [8,10,11,12].

Among all microscopic findings, the ganglion-like cells remain the single most distinctive clue to the diagnosis. Even so, their value depends on the company they keep. Similar polygonal cells have occasionally been described in other reactive and neoplastic conditions, making them insufficient as an isolated diagnostic criterion. In proliferative fasciitis, they occur within a characteristic fibroblastic and myofibroblastic proliferation that provides the context necessary for their interpretation. This relationship is easily underestimated when only limited tissue is available, explaining why small biopsies may present a greater diagnostic challenge than complete excision specimens [8,12,13].

Mitotic activity deserves similar consideration. Early reports already emphasized that mitotic figures are not uncommon in proliferative fasciitis and should not, by themselves, suggest malignancy. Subsequent publications have reached the same conclusion, consistently describing mitoses as typical rather than atypical. Our findings are entirely in keeping with these observations. Although mitotic figures were readily identified, they remained morphologically normal and were not accompanied by diffuse cytological atypia or tumour necrosis, features that would be expected in a genuinely malignant spindle-cell proliferation [8,12,14].

The changes observed at the periphery of the lesion were equally informative. Irregular extension into adjacent adipose tissue, together with focal fat necrosis and a granulomatous histiocytic response, initially created an impression of locally aggressive growth. Closer examination suggested a different interpretation. The transition between the lesion and the surrounding tissue remained gradual, without destructive invasion or progressive replacement of pre-existing structures. In this context, the peripheral changes were more compatible with a reactive tissue response than with infiltrative behaviour. These observations formed the basis for the practical approach summarized in Table 1.

Rather than listing favourable and unfavourable microscopic findings, the table separates those features that tend to attract immediate attention from those that proved most useful when the lesion was assessed as a whole. The distinction is subtle, but it reflects the sequence in which difficult cases are often resolved in routine surgical pathology.

The differential diagnosis of proliferative fasciitis is shaped as much by clinical setting as by microscopic appearance. Although several spindle-cell lesions may share overlapping histological features, they do not enter the diagnostic consideration for the same reasons, nor do they remain equally plausible throughout slide evaluation. The differential diagnosis therefore evolves as additional information becomes available, with some entities becoming progressively less likely as the lesion is examined in its entirety [5,6,8,15,16].

Among benign lesions, nodular fasciitis remains the closest histological counterpart. The relationship between the two entities has long been recognized, and current molecular studies have further emphasized that they represent biologically distinct lesions despite substantial morphological overlap. While recurrent USP6 rearrangements define nodular fasciitis, proliferative fasciitis and proliferative myositis are characterized in many cases by *FOS* rearrangements, supporting the concept that they occupy a separate position within the spectrum of fibroblastic and myofibroblastic proliferations. From a practical perspective, however, this distinction has not diminished the importance of conventional histopathology. Most diagnoses continue to rely on routine microscopic examination because molecular testing is neither universally available nor required in typical cases [5,15,16].

The distinction from proliferative myositis is usually less problematic once the anatomical compartment is recognized. Cytologically, the two lesions are remarkably similar and likely represent closely related processes. Their separation rests primarily on anatomical distribution. Proliferative myositis characteristically permeates skeletal muscle, producing the well-known checkerboard pattern, whereas proliferative fasciitis develops in close association with the superficial fascia and subcutaneous tissue. In the present lesion, the absence of skeletal muscle involvement effectively excluded proliferative myositis despite the close resemblance at higher magnification [5,9,17].

The more demanding distinction is with spindle-cell sarcomas, particularly lesions showing myofibroblastic differentiation. Unlike reactive proliferations, these neoplasms usually display a greater degree of internal uniformity. Once atypical cytological features appear, they tend to persist throughout the lesion rather than alternating with areas of more conventional reactive morphology. Their growth also becomes progressively infiltrative instead of remaining centred within a relatively localized proliferation. None of these characteristics was identified in our specimen. The histological appearance varied from one area to another, yet the lesion retained an overall internal consistency that favoured a benign fibroblastic and myofibroblastic proliferation [5,6,18,19].

Immunohistochemistry supported, but did not establish, the diagnosis in the present case. The observed immunophenotype, characterized by focal SMA expression, weak focal desmin positivity, and a low Ki-67 proliferative index, was consistent with previously reported cases of proliferative fasciitis and supported myofibroblastic lineage. Equally informative was the absence of CD34 and CD31 expression, which helped narrow the differential diagnosis while remaining compatible with the expected immunophenotype of proliferative fasciitis. Nevertheless, these findings were interpreted in conjunction with the characteristic morphology and clinical setting rather than as independent diagnostic criteria [8,9,12].

The focal S100 positivity observed in the present case deserves specific comment because it may initially broaden the differential diagnosis toward neural or melanocytic lesions. However, the staining was weak, focal, and predominantly confined to ganglion-like cells, without the diffuse expression expected in peripheral nerve sheath tumors. Occasional focal S100 expression has been reported in proliferative fasciitis and should therefore be interpreted cautiously within the overall clinicopathological context rather than in isolation [20,21].

The immunohistochemical panel was selected on the basis of the morphological differential diagnosis generated after routine histopathological evaluation. Rather than relying on an extensive exclusion panel, the selected markers were used to support the morphological impression and to exclude the most relevant diagnostic mimics suggested by the histological findings.

Table 2 summarizes the practical observations that proved most helpful during case evaluation and identifies the findings that ultimately favoured each competing diagnosis or argued against it. Although simplified, this approach more closely reflects routine diagnostic practice than a checklist of isolated histological characteristics.

The increasing availability of molecular diagnostics has undoubtedly refined the classification of fibroblastic and myofibroblastic proliferations, providing new insights into lesions that were traditionally regarded as purely reactive. Nevertheless, the practical approach to proliferative fasciitis has changed surprisingly little. In most pathology laboratories, diagnosis continues to rely on the integration of clinical information with routine histopathological examination, while ancillary molecular techniques remain reserved for selected cases in which conventional assessment fails to provide a satisfactory explanation. Our findings reflect this reality. Although molecular confirmation was not available, the clinicopathological and histological features were sufficiently characteristic to establish the diagnosis with confidence [22,23,24,25].

**Figure 5:**
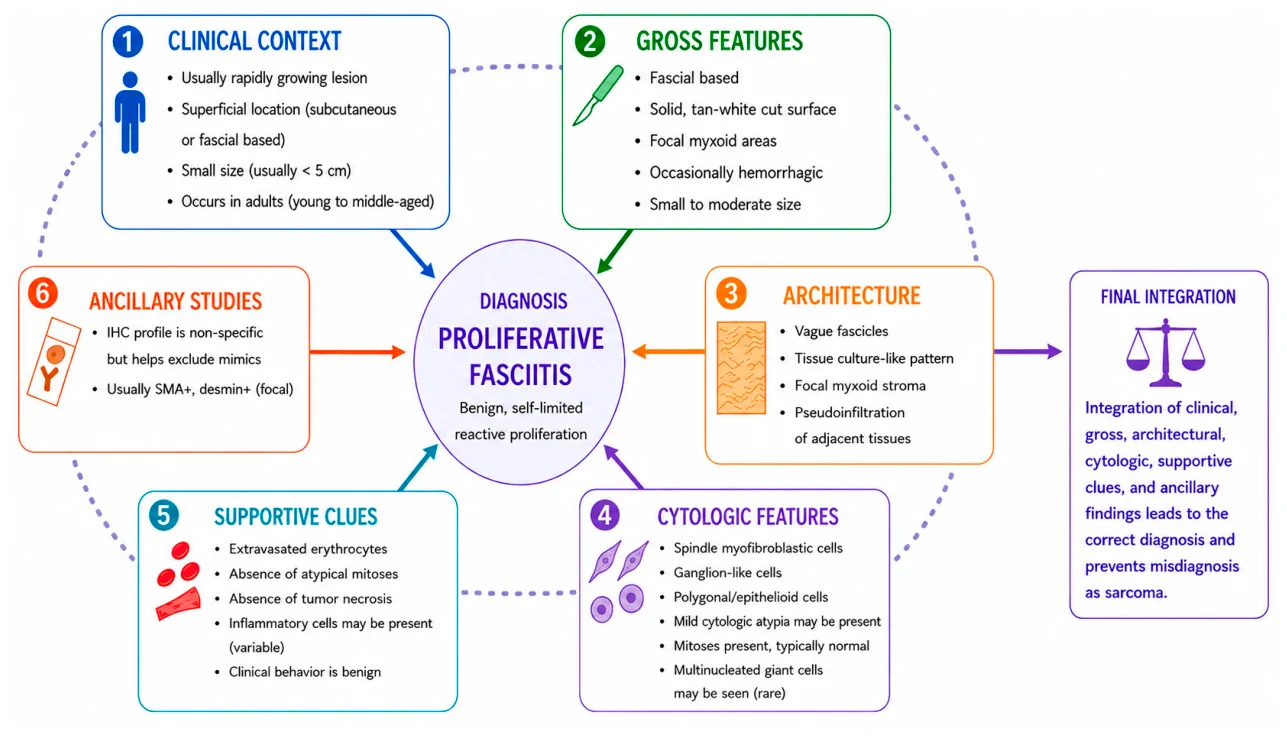
Proposed clinicopathological framework for the evaluation of proliferative fasciitis and its principal differential diagnoses. The framework summarizes the diagnostic considerations derived from the present case and the published literature and is intended to facilitate systematic clinicopathological assessment of diagnostically challenging superficial spindle-cell proliferations. It was developed by the authors as an educational synthesis of clinicopathological features and has not undergone independent expert validation or formal diagnostic performance assessment. It should therefore not be interpreted as a validated diagnostic algorithm or as a substitute for comprehensive pathological evaluation.

The clinicopathological framework proposed in Figure 5, together with the practical considerations summarized in Table 1 and Table 2, was developed with this objective in mind. Rather than introducing new diagnostic criteria, these tools translate clinicopathological knowledge into a structured approach that can be applied during routine slide evaluation. They are intended to complement existing diagnostic descriptions by emphasizing the sequence in which observations are assessed and integrated, particularly in lesions that initially appear more aggressive than their biological behaviour would suggest (Figure 5).

This report has several limitations that should be acknowledged. First, molecular confirmation by *FOS* rearrangement analysis or FOS immunohistochemistry was not available. Although these ancillary techniques may provide additional diagnostic support in selected diagnostically challenging cases, current evidence indicates that the diagnosis of proliferative fasciitis continues to rely primarily on clinicopathological correlation. Second, preoperative imaging was not performed because the lesion lacked clinical features suggestive of malignancy and was considered suitable for primary excision at the initial presentation. Consequently, radiological-pathological correlation could not be assessed. The current follow-up period remains relatively short and does not permit definitive assessment of long-term clinical outcome, although no evidence of local recurrence has been observed to date. Finally, no formal inter-observer reproducibility assessment of the histomorphological features or mitotic count was performed, as this single-case report was not designed as a diagnostic reproducibility study.

## 4. Conclusions

Although uncommon, proliferative fasciitis continues to represent a significant diagnostic pitfall because its microscopic appearance may appear disproportionately alarming when compared with its benign clinical behaviour. Careful clinicopathological correlation, supported by histopathological examination and appropriately selected immunohistochemistry, remains the cornerstone of diagnosis. Recognizing this spectrum is essential to distinguish proliferative fasciitis from its malignant mimics and to prevent unnecessary aggressive management.

## Figures and Tables

**Table 1:** Practical morphological clues for distinguishing proliferative fasciitis from its malignant mimics. The table summarizes the most frequent diagnostic pitfalls encountered during microscopic evaluation of proliferative fasciitis and provides practical considerations for distinguishing this benign pseudosarcomatous proliferation from malignant spindle cell neoplasms.

Potential Diagnostic Pitfall	Why It May Be Misleading	Practical Clue Favoring Proliferative Fasciitis
Marked hypercellularity	May suggest spindle cell sarcoma	Assess low-power architecture; overall growth pattern is more informative than cellular density alone.
Frequent mitotic figures	May suggest malignancy	Typical mitoses without atypical forms favor PF; mitotic quality is more informative than mitotic count.
Cytological pleomorphism	May suggest pleomorphic sarcoma	Usually focal and limited; diffuse marked atypia should prompt reconsideration.
Ganglion-like/polygonal cells	May mimic epithelioid or pleomorphic neoplasms	Interpret with the accompanying spindle-cell myofibroblastic proliferation.
Pseudoinfiltrative extension into adipose tissue	May mimic destructive infiltration	Correlate with overall growth pattern and absence of destructive invasion.
Myxoid stromal change	May suggest myxoid sarcoma	Interpret with tissue culture-like areas, extravasated erythrocytes, and overall morphology.
Osteoclast-like multinucleated giant cells	May raise concern for undifferentiated pleomorphic sarcoma	Uncommon but recognized in PF; interpret within the overall morphological context.
Non-specific immunohistochemical profile	May increase diagnostic uncertainty	Supports diagnosis and helps exclude mimics but should not replace morphological assessment.
Reliance on a single microscopic feature	May lead to over- or underdiagnosis	PF requires clinicopathological integration; no isolated feature is diagnostic.

**Table 2:** Principal differential diagnoses of proliferative fasciitis in routine surgical pathology. Reactive and neoplastic spindle-cell lesions that may resemble proliferative fasciitis, together with their typical clinical setting and the clinicopathological findings that most strongly favor or argue against each diagnosis.

Entity	Typical Clinical Setting	Anatomical Location	Clinicopathological Features Favouring the Alternative Diagnosis	Findings Supporting Proliferative Fasciitis	Representative References
Nodular fasciitis	Usually rapidly growing superficial nodule in young to middle-aged adults	Subcutaneous tissue, fascia	Lacks ganglion-like cells; characteristic tissue culture-like growth pattern; USP6 rearrangement when tested	Presence of ganglion-like cells; biphasic spindle-cell and ganglion-like cell population	[3,15,16]
Proliferative myositis	Rapidly enlarging intramuscular mass	Skeletal muscle	Characteristic checkerboard entrapment of skeletal muscle fibers	Lesion confined to superficial fascia or subcutaneous tissue without skeletal muscle involvement	[4,5]
Low-grade myofibroblastic sarcoma	Slowly enlarging deep soft tissue mass	Deep soft tissues of head and neck or extremities	Infiltrative growth with diffuse cytological atypia and infiltrative margins	Limited atypia, reactive architectural heterogeneity, typical mitoses	[18,19]
Inflammatory myofibroblastic tumor	Soft tissue or visceral mass, sometimes accompanied by inflammatory symptoms	Deep soft tissues or visceral organs	Prominent inflammatory infiltrate; ALK or other kinase alterations may be present	Minimal inflammation; ganglion-like cells; superficial fascial location	[20,21]
Myxofibrosarcoma	Slowly enlarging extremity mass, usually in older adults	Deep dermis and subcutaneous tissue	Diffuse myxoid stroma with characteristic curvilinear vessels and infiltrative growth	Only focal myxoid change; absence of diffuse infiltrative growth; ganglion-like cells present	[1,21]
Undifferentiated pleomorphic sarcoma	Rapidly enlarging deep soft tissue tumor	Deep soft tissues of extremities	Marked pleomorphism, atypical mitotic figures, and tumor necrosis	Mild focal pleomorphism only; typical mitoses; absence of tumor necrosis; reactive architecture	[1,2]
Desmoid fibromatosis	Slowly growing firm fascial mass	Deep musculoaponeurotic structures	Long sweeping fascicles; infiltrative borders; nuclear β-catenin expression	Greater cellular heterogeneity; ganglion-like cells; myxoid change; reactive clinicopathological features	[2,20]

## Data Availability

The original data presented in the study are included in the article; further inquiries can be directed to the corresponding author.
